# Assessment of Rival Males through the Use of Multiple Sensory Cues in the Fruitfly *Drosophila pseudoobscura*

**DOI:** 10.1371/journal.pone.0123058

**Published:** 2015-04-07

**Authors:** Chris P. Maguire, Anne Lizé, Tom A. R. Price

**Affiliations:** 1 Institute of Integrative Biology, University of Liverpool, Liverpool, United Kingdom; 2 UMR 6553 ECOBIO, Université de Rennes 1, Rennes, France; Universidad Nacional Autonoma de Mexico, MEXICO

## Abstract

Environments vary stochastically, and animals need to behave in ways that best fit the conditions in which they find themselves. The social environment is particularly variable, and responding appropriately to it can be vital for an animal’s success. However, cues of social environment are not always reliable, and animals may need to balance accuracy against the risk of failing to respond if local conditions or interfering signals prevent them detecting a cue. Recent work has shown that many male *Drosophila* fruit flies respond to the presence of rival males, and that these responses increase their success in acquiring mates and fathering offspring. In *Drosophila melanogaster* males detect rivals using auditory, tactile and olfactory cues. However, males fail to respond to rivals if any two of these senses are not functioning: a single cue is not enough to produce a response. Here we examined cue use in the detection of rival males in a distantly related *Drosophila* species, *D*. *pseudoobscura*, where auditory, olfactory, tactile and visual cues were manipulated to assess the importance of each sensory cue singly and in combination. In contrast to *D*. *melanogaster*, male *D*. *pseudoobscura* require intact olfactory and tactile cues to respond to rivals. Visual cues were not important for detecting rival *D*. *pseudoobscura*, while results on auditory cues appeared puzzling. This difference in cue use in two species in the same genus suggests that cue use is evolutionarily labile, and may evolve in response to ecological or life history differences between species.

## Introduction

Animals often show rapid behavioural and physiological changes to survive and reproduce in a changeable environment [1, 2]. In particular, the socio-sexual environment can change very quickly, and is therefore extremely important for male mating success [1]. Competition between males for access to females is known to generate aggressiveness between males in many animals such as mice [3], birds [4], fish [5], spiders [6], and flies [7, 8]). Expressing aggressiveness toward a conspecific depends on the ability to recognize and identify individuals within a social context [9]. For example, a male that is unable to recognize other males or distinguish them from females may waste energy by attempting to court both males and females alike. In recognition systems (social, mate, kin or family, predatory etc.), an individual has to express or bear a cue that will be perceived and processed by receivers, who will respond (or not) to it appropriately.

Recognition systems are costly as any errors may lead to reduced fitness both for the emitter and the receiver of the cue [10–12]. Therefore recognition systems are generally very specific and sometimes highly complex. In some species, single environmental cues are used as key indicators of sperm competition risk [13]. For example, the male meadow vole (*Microtus pennsylvanicus*) will increase its sperm investment after detecting the risk of sperm competition through the odour of conspecific males [14]. However, in many systems multiple cues are likely to be used, and how multiple cues are perceived and integrated by the receiver to generate a response is poorly understood. Indeed, understanding how multiple cues are integrated over both behavioural and evolutionary time is a key area of research in behavioral ecology, in behaviors including mate choice and struggles for dominance [15,16], investment in offspring and rejection of brood parasites [17] and habitat selection [18]. As experiments on detection of rivals and responses to them can be carried out in the laboratory [1], they provide a potentially enlightening area in which to examine the integration of cues. However, at present, there are few studies that assess the use of multiple cues to effectively respond to rivals [1]. In addition, the fitness consequences of such physiological and behavioural responses have received very little attention in the literature [1]. In light of this significant gap in knowledge, two studies using *Drosophila*, have tried to identify the potential multiple cues that males use to assess the risk of sperm competition and to examine the fitness effects of the resultant plastic behaviours [13, 19].

However, recognizing a competitor, a mate or a potential predator does not necessarily mean that the individual will express a response. In fact, we can only behaviorally detect recognition systems and assess them when the receiver expresses a response to the emitter (i.e. when an interaction occurs). This notion suggests that an absence of behaviour as a response does not necessarily mean that recognition did not occur. This is particularly difficult to deal with as we cannot ascertain that recognition was absent when there is no interaction between individuals. Deciphering the cues used, and how they are processed by receivers, is essential as it allows us to some extent to study recognition systems.

Male behavioural plasticity has been shown in numerous *Drosophila* species, as a response to the threat of sperm competition [19–21]. In the presence of rivals, males will undergo prolonged copulation and show an increase in a latency to copulate [21]. *D*. *pseudoobscura* has been shown to effectively alter the proportion of eusperm (fertilising) and parasperm (non-fertilising) in their ejaculate [22]. Additionally, *D*. *melanogaster* have been shown to strategically allocate the proportions of their seminal fluid proteins, in response to the risk of sperm competition [23], and to increase the number of sperm in their ejaculate after exposure to a rival male [24]. These variable responses made to rivals have subsequently been shown to cause a gain in fitness in both *D*. *melanogaster* and *D*. *pseudoobscura*, through a direct increase in offspring [20, 22]. However, in *D*. *melanogaster* this response to potential rivals has fitness costs in terms of male survival and mating success later in life, making it important that males respond appropriately to rivals [25].

The ability to assess potential rivalry in male *Drosophila* suggests a highly accurate mechanism for perception, allowing behavioural plasticity to be performed to a high degree of subtlety. A recent study has shown that *D*. *melanogaster* males use a combination of tactile, olfactory and auditory cues to perceive the threat of sperm competition and attune their plastic behavioural responses accordingly [13]. To identify the cues used, Bretman et al. [13], systematically removed all possible cues of vision, touch, smell and sound from a male. They then applied each sensory manipulation, in all possible combinations, and measured the ability of the treated male to identify rivals (i.e. the potential for sperm competition). They report that a combination of any two sensory cues from smell, sound or touch can be used, interchangeably, to allow the perception of rival males in *D*. *melanogaster*. Visual cues were determined to be of only marginal importance.

Despite the significance of these results, this work has neither been fully replicated nor has it been repeated in any other species. A recent study has found similar results for the importance of olfactory and auditory cues, although they also used *D*. *melanogaster* [24]. Here, we undertake a retesting of the Bretman *et al*. [13] study using *D*. *pseudoobscura*, a species only distantly related to *D*. *melanogaster* [26]. *D*. *pseudoobscura* has been shown to exhibit similar plastic responses to mate rivalry to those observed in *D*. *melanogaster*, i.e., males have been shown to increase their copulation duration in response to the risk of sperm competition [22]. Therefore, we examine whether *D*. *pseudoobscura* also identify male rivals using the same combinations of sensory cues.

## Methods

Wild *D*. *pseudoobscura* females were collected at Show Low, Arizona, USA (34.205413°N, 109.941420°W) in 2008. No specific permissions were required to collect at this location as it was a National Forest, which was confirmed by Lakeside Ranger Station, and *D*. *pseudoobscura* is not endangered or protected, instead being extremely common. The offspring of each wild caught female were inbred over several generations to create isofemale lines, which maintain genetic diversity as their high homozygosity prevents adaptation to the laboratory environment [27]. Twenty of these lines were then crossed to produce an outbred population in 2011. All isolines used carried the normal X chromosome (referred to as "*Standard*" or "*ST*"), which shows normal Mendelian inheritance. However, some *D*. *pseudoobscura* carry the meiotic driving X chromosome "*Sex-Ratio*" or "*SR*". In males, *SR* causes the death of all Y chromosome sperm, resulting in all female broods [28]. We used this to generate large numbers of females for use in our experiments. A strain of *SR* was isolated from the collected flies in 2008. In 2011 it was introgressed into the *ST* population background by repeated crosses, resulting in flies that had the same outbred background as the *ST* population, but carried *SR* rather than *ST*. Males for the experiment were collected from the *ST* population. Females were taken from *SR* males crossed to *SR*/*SR* females, a cross which produces only daughters and infertile pseudomales, making the collection of virgin females very simple.

Populations were reared and maintained in a 22°C humidified room under a 14:10h, light:dark photoperiod (lights on at 10:00am GMT, Liverpool UK). Flies were kept in standard *Drosophila* vials (75 x 25 mm) with 10ml of a sugar-yeast medium (10g agar, 85g sugar, 60g maize, 40g yeast, 1000ml water and 25ml nipagin (10% w/v solution) per litre). Experimental flies were collected within 18 h of eclosion, to ensure the males were virgin, with sexes separated by aspiration [28]. Females were isolated and placed in vials at a density of 10 per vial and allowed to mature for four days (adapted from [21]). Only *SR/SR* genotype females were used during the experimental tests.

*ST* males were collected upon eclosion and isolated into individual vials. Males were then randomly allocated to one of 26 treatments (See Table 1 for treatment descriptions). All treatments were produced by removing the production or perception of sensory cues of rival or focal males respectively within 24 hours of their eclosion. Treatment manipulations followed similar methods used by Bretman *et al*. [13]. However, it was not possible to conduct genetic removal of cues, by using lines of mutant flies that had lost the ability to perceive olfactory, visual and auditory cues (as used by Bretman *et al*. [13]), as such mutants were not available in *D*. *pseudoobscura*. Cues were removed phenotypically, one sense at a time and then in combination to produce all treatments described (See Table 1). All surgical removal performed on flies was carried out under CO_2_ anaesthesia, using humidified CO_2_ through a standard *Drosophila* gas stage [29]. The removal of auditory cues was achieved by surgically removing both wings of the rival male. This was done using a scalpel to cut at the wing base, removing the entire wing structure on both sides. This stopped the production of the courtship song, which is reported to be the essential component of auditory cues in *D*. *melanogaster* [13]. To remove olfactory cues the third segment of the focal male antenna were sliced off with sharpened forceps. This also removes the aristae, which are used in the detection of sound. However, it has been shown in a previous experiment that not all sound perception is lost upon aristae removal [13]. Visual cues were removed by placing males in complete darkness. Finally, to remove tactile cues the focal and rival males were placed into two separate vials with the vial openings placed together but separated by porous netting, allowing visual, olfactory and auditory cues, but preventing touch.

**Table 1:** Summary of the sensory cue(s) removed for each treatment, with the respective control treatment used for comparisons set out on the same line.

Treatments	N	Description	Controls	N	Description
C	119	Control	CC	42	Control—CO_2_ anaesthesia
CNR	123	Control (no rival)	CCNR	40	Control (no rival)—CO_2_ anaesthesia
A	83	Auditory removal	CNR	123	Control (no rival)
O	76	Olfactory removal	ONR	81	Olfactory removal (no rival)
T	76	Tactile removal	TNR	81	Tactile removal (no rival)
V	82	Vision removal	VNR	82	Vision removal (no rival)
AO	72	Auditory + Olfactory removal	CNR	123	Control (no rival)
AV	80	Auditory + Vision removal	CNR	123	Control (no rival)
AT	80	Auditory + Tactile removal	CNR	123	Control (no rival)
OV	77	Olfactory + Vision removal	OVNR	72	Olfactory + Vision removal (no rival)
OT	39	Olfactory + Tactile removal	OTNR	42	Olfactory + Tactile removal (no rival)
TV	78	Tactile + Vision removal	TVNR	77	Tactile + Vision removal (no rival)
AOV	82	Auditory + olfactory + vision removal	CNR	123	Control (no rival)
ATV	74	Auditory + tactile + vision removal	CNR	123	Control (no rival)
AOT	38	Auditory + Olfactory removal	CNR	123	Control (no rival)
OTV	39	Olfactory + Tactile + Vision removal	OTVNR	44	Olfactory + Tactile + Vision removal (no rival)
AOTV	42	Auditory + Olfactory + Tactile + Vision removal	CNR	123	Control (no rival)

Note that some control treatments are listed more than once in the table as they act as controls for more than one experimental treatment.

After sensory manipulation, a focal male was either conditioned with a rival male or placed alone in a vial (with no rival) for four days to acclimatise, producing the 26 treatments (Table 1) [22]. This provided internal controls for each treatment which allowed the effects of sensory cue loss to be tested both in the presence and absence of a rival male. Rival males were distinguished from focal males by the removal of one of their wings under CO_2_ anaesthesia. This procedure was followed for each treatment except for those that involved the removal of the auditory cue, as this set of treatments already required the removal of both wings from the rival. Therefore, the treatment in which males were conditioned alone was used as the internal control for auditory cue treatments (CNR). Furthermore, the influence of CO2 anaesthesia on males was estimated by comparing aspirated (non anaesthetized) males stored with one rival (C) to CO2 anaesthetized males stored with one rival (CC). In the same way, aspirated (non anaesthetized) males stored singly (CNR) were compared to CO2 anaesthetized males stored singly (CCNR).

Three-day old females were removed from their groups of 10 and placed into individual standard vials, then left overnight to acclimatise. Each focal male was then moved by aspiration into a randomly allocated female vial. Rival males remained in their vial until the focal male was removed, at which point the rival male was discarded. Upon introduction of the focal male to the vial, copulation latency (time between the male being placed in the vial to the onset of copulation), and copulation duration (time elapsed from genital contact to detachment) were measured. Experiments were carried out between 11:00am and 14:00 GMT in February 2013, at Liverpool, UK. Females were classed as unmated if copulation was not observed after two hours. Copulation durations longer than 10 minutes or shorter than 1 minute (‘pseudo-copulations’; [30]) were considered as outliers and consequently removed from the analysis to ensure normality. This resulted in 76 data points out of a total 1883 (a typical rate of pseudocopulations for this population, Pers. Comm., T. Price).

### Statistical analysis

All statistical analyses were performed using R 3.1.0 [31]. Copulation latencies and copulation duration of treated males responding to rival presence were compared with copulation latencies and copulation duration of males (treated or not) stored in the absence of rival (control treatments). Data were not normally distributed and transformed by a Box-Cox procedure to obtain normally distributed residuals [32]. Following Box-Cox transformations, each treatment was compared to its control treatment (see Table 1 for details) by a Welch’s two sample t-tests, which correct for variance differences among treatments.

## Results

### Effect of the presence of a rival male

The control treatments, in which no cues were removed, showed the same pattern of changes in copulation duration and latency as previously reported for the species [22]. Males exposed to a rival subsequently copulated for significantly longer (CC-CCNR: t = 4.2676, df = 79.456, p-value = 5.415^e-5^), but showed no difference in the latency to copulate (CC-CCNR: t = 1.1660, df = 75.201, p-value = 0.2473).

### Effect of carbon dioxide anaesthesia

**Fig 1:**
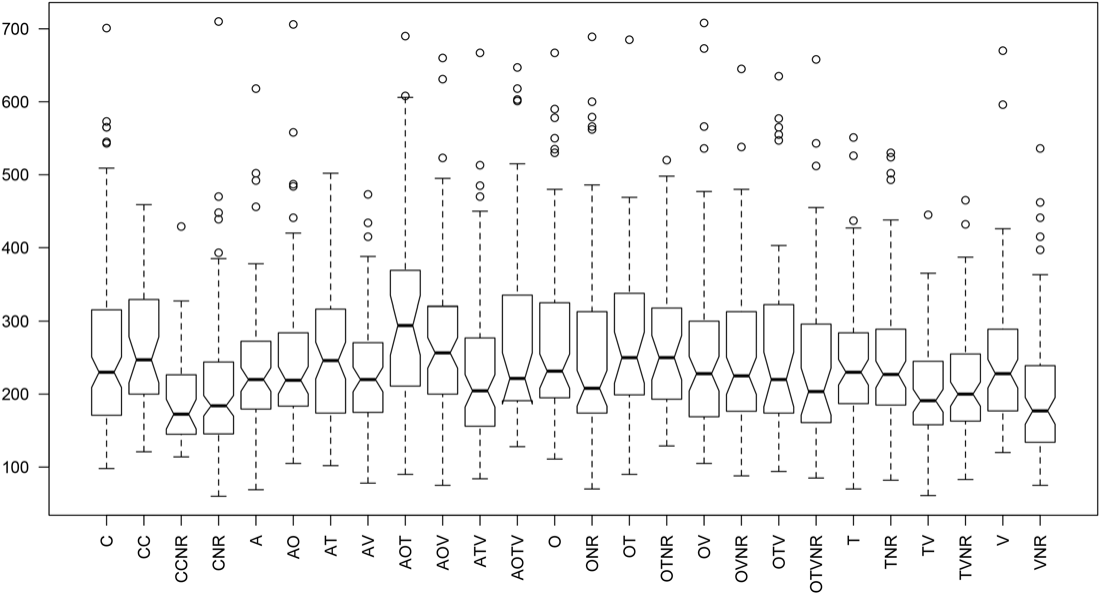
Notched boxplot of copulation durations represented by the median (black lines), indicating 95% confidence interval of medians (the notches), interquartile ranges (upper and lower limits of the notches), and the minimum and maximum values (lower and upper whiskers). For the details of treatments see Table 1.

**Fig 2:**
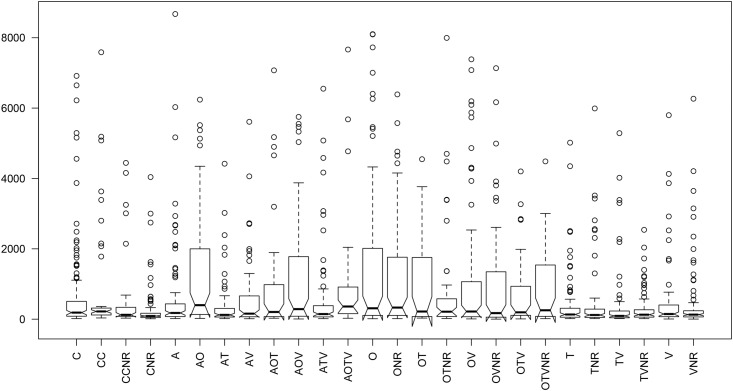
Notched boxplot of copulation latencies represented by the median (black lines), indicating 95% confidence interval of medians (the notches), interquartile ranges (upper and lower limits of the notches), and the minimum and maximum values (lower and upper whiskers). For the details of treatments see Table 1.

Carbon dioxide (CO_2_) anaesthesia had no effect on copulation latency of males not exposed to rivals (C-CC: t = -0.7078, df = 61.652, p-value = 0.4817). However, males exposed to rivals tend to increased copulation latency when they have previously been anaesthetized by CO_2_ (CNR-CCNR: t = -1.9817, df = 46.419, p-value = 0.05345) (Fig 1). CO_2_ had no significant effect on copulation duration (C-CC: t = -0.5456, df = 98.551, p-value = 0.5866; CNR-CCNR: t = 0.6318, df = 84.519, p-value = 0.5292) (Fig 2).

### Copulation duration

Copulation duration was not altered in the presence of a rival male when either the Tactile (T-TNR: t = -0.0793, df = 154.206, p-value = 0.9369), or Olfactory (O-ONR: t = -1.1611, df = 154.815, p-value = 0.2474) cue was removed. However, when the Visual cues were removed, male copulation duration was still significantly longer after exposure to a rival (Fig 2; V-VNR: t = -3.142, df = 161.843, p-value = 0.001996). This means that tactile and olfactory cues are essential for males to detect rival and adapt copulation duration, while visual cues are not. This pattern continued when two cues were removed. Removal of Olfactory + Tactile (OT-OTNR: t = -0.2618, df = 74.527, p-value = 0.7942), Olfactory + Visual (OV-OVNR: t = -0.2833, df = 146.322, p-value = 0.7773), and Tactile + Visual (TV-TVNR: t = 1.1379, df = 150.843, p-value = 0.2570) led to no significant change in copulation duration when a rival was present. When all three Olfactory + Tactile + Visual cues were removed, again males did not alter their copulation duration after exposure to a rival (OTV-OTVNR: t = -1.2011, df = 77.044, p-value = 0.2334).

However, when Auditory cues were removed, the pattern was much more complex. Focal males increased their copulation duration after exposure to a rival when Auditory cues had been removed (A-CNR: t = -2.2294, df = 175.743, p-value = 0.02705). Focal males exposed to rivals also showed longer copulation duration when Auditory + Tactile (AT-CNR: t = -3.8392, df = 169.78, p-value = 0.0001), Auditory + Olfactory (AO-CNR: t = -3.5198, df = 140.85, p-value = 0.0005), or Auditory + Visual (AV-CNR: t = -2.1693, df = 178.802, p-value = 0.0313) cues were removed (Fig 2), despite the removal of Tactile or Olfactory cues preventing a response to rivals when each was removed alone. In the treatments where Auditory was one of three cues removed, males did not alter copulation duration after exposure to a rival when Auditory + Tactile + Visual (ATV-CNR: t = -1.4845, df = 136.346, p-value = 0.1400) cues were removed, but did when Auditory + Olfactory + Tactile (AOT-CNR: t = -4.5681, df = 49.293, p-value = 3.312^e-5^), or Auditory + Olfactory + Visual (AOV-CNR: t = -4.7289, df = 157.808, p-value = 4.971^e-6^) cues were removed (Fig 2). Furthermore, males had longer copulation duration after exposure to rivals when all four cues had been removed (AOTV-CNR: t = -3.4484, df = 55.306, p-value = 0.0010) (Fig 2).

### Copulation latency

Males did not alter their copulation latency when exposed to a rival if Tactile (T-TNR: t = 0.087, df = 154.986, p-value = 0.9308), Olfactory (O-ONR: t = 0.9187, df = 141.772, p-value = 0.3598), or Visual (V-VNR: t = 0.0098, df = 161.285, p-value = 0.9922) cues were removed. When two cues were removed, the combined removal of Olfactory + Tactile (OT-OTNR: t = 0.5257, df = 78.999, p-value = 0.6006), or Tactile + Visual (TV-TVNR: t = 0.3346, df = 129.532, p-value = 0.7385), or Olfactory + Visual (OV-OVNR: t = 0.7556, df = 144.875, p-value = 0.4511) cues also prevented males from altering their copulation latency after exposure to rivals, as did the removal of all three Olfactory + Tactile + Visual cues (OTV-OTVNR: t = -0.6512, df = 80.422, p-value = 0.5168).

However, the removal of Auditory cues again resulted in a more complex pattern of changes in response to the presence of rivals. Removing Auditory cues alone (A-CNR: t = -3.3953, df = 106.743, p-value = 0.0009) lead to a longer copulation latency after exposure to a rival, meaning that auditory cues were not essential to detect rivals (Fig 1). Removing both Auditory + Tactile (AT-CNR: t = -1.6471, df = 142.042, p-value = 0.1017) cues resulted in no difference in copulation latency between rival exposed and naïve males. However, when Auditory + Olfactory (AO-CNR: t = -5.9879, df = 84.198, p-value = 5.047^e-8^), or Auditory + Visual (AV-CNR: t = -3.0444, df = 120.818, p-value = 0.002862) cues were removed, males showed longer copulation latency when exposed to a rival (Fig 1). Similarly, males exposed to rivals showed longer copulation latency when Auditory + Olfactory + Tactile (AOT-CNR: t = -2.9976, df = 40.374, p-value = 0.004639), Auditory + Olfactory + Visual (AOV-CNR: t = -5.6141, df = 99.322, p-value = 1.797^e-7^), or Auditory + Tactile + Visual (ATV-CNR: t = -2.756, df = 95.424, p-value = 0.00701) cues were removed (Fig 1), or when all four cues were removed (AOTV-CNR: t = -3.4114, df = 46.503, p-value = 0.001347).

## Discussion

Our results for tactile, olfactory and visual cues showed a broadly consistent pattern. The typical increase in copulation duration shown by males exposed to a rival did not occur when olfactory or tactile cues were removed. This happened if either olfactory or tactile cues were the only ones removed, or if they were removed in any combination with each other or visual cues. The removal of any one of tactile, olfactory, or visual cues was enough to prevent a change in copulation latency when previously exposed to a rival. However, treatments involving auditory cue removal showed a far less clear pattern. Males, whose auditory cues had been removed, still responded to rivals in both copulation latency and duration. However, auditory cues had an unexpected effect when they were removed in combination with the loss of tactile or olfactory cues. The loss of tactile or olfactory cues normally prevented a male from increasing his copulation duration when exposed to a rival, but if a male had also lost his auditory cues, then that male was able to show the increase in copulation duration. This is puzzling, because if removing olfactory cues prevented a response to rivals, why would the removal of both olfactory and auditory cues allow rivals to be detected?

There are several potential explanations for the puzzling results of the auditory trials. One possibility is that removal of the rival wings in the auditory treatments might have altered the behaviour of the rival male, making it more detectable than an intact rival male. Indeed, wing removal might have stressed the rival males, which might then have moved more rapidly around the vial, encountering the focal male more often and creating a stronger signal that a rival male is present. A second possibility is that the scent of a damaged male is easier to detect. Alternatively, wing removal may have prevented auditory male-male threat signals by the wingless rival. *Drosophila* males may use loud “wing buzz” signals to deter other males [33]. A wingless rival is unable to use these signals, and so might escalate to physical attacks more frequently, making the presence of a wingless rival more detectable than a normal winged rival. Increases in copulation duration after exposure to a rival in the monandrous species *D*. *subobscura* have been ascribed to the impact of physical attacks between males [34], so this is a possibility. However, these hypotheses cannot explain the increase in copulation duration after exposure to a rival seen in the treatment where all auditory, olfactory, tactile and visual cues were removed. This suggests that there may be an additional cue which we did not investigate, or that our removal of cues was not entirely effective.

A more parsimonious explanation might be that a combination of chance and experimental error may have created the significant increases in copulation duration when auditory signals were removed in combination with other cues. The auditory treatments were unusual because they could not be compared to direct "no-rival" controls as the perception of auditory cues was manipulated by removing the wing of the rival male: when the rival was absent, there was no auditory treatment. Thus for the auditory treatments, the control used for comparisons was simply the treatment where the rival male was absent. Copulation duration was particularly short for CNR males and this might have exacerbated the differences found between the auditory treatments and their controls. For all of these potential reasons, our manipulation of auditory cues may have had unexpected impacts, or simply be flawed, and the results must be interpreted cautiously. Ideally, the experiment should be repeated using deaf focal males, as was carried out by Bretman *et al*. [13] using mutants available in *D*. *melanogaster*, but currently unavailable for *D*. *pseudoobscura*.

Another area of concern for this experiment is whether a non-significant change in copulation duration between treatment and control really indicates that the rival could not be detected in that treatment. Although we used the standard biological p value significance limit (p<0.05 indicates significance), it does not follow that p values higher than 0.05 prove that there is no difference in behaviour between treatments. P values higher than 0.05 only indicate that the difference is not significant in the sample of trials observed. This is particularly relevant when assessing the effect of cue removal on the absence of behavioural response. As highlighted in the introduction, we cannot necessarily conclude that recognition did not occur simply because we did not observe a behavioural change. The study of recognition systems is limited by this, and will always be, as long as recognition is measured indirectly [35], rather than by studying direct neuronal and/or physiological responses. The second weakness of our study concerns copulation latency. As we did not detect any differences in copulation latency between our intact control males that were exposed to rivals, and our intact control males that were not exposed to rivals, this suggests that we should not have analysed the effect of rivals on copulation latency for the remaining treatments. However, a previous study found that exposure to a rival male did alter copulation latency in *D*. *pseudoobscura* [21,34]. The changes that occur in copulation latency in response to rivals are not well understood in *Drosophila*. One expectation might be that males exposed to rivals should invest more in attempting to find and court mates, resulting in reduced copulation latency after exposure to a rival. However, male *D*. *melanogaster* [20] and *D*. *acanthoptera* [21] show no change in copulation latency when exposed to a rival. In contrast, male *D*. *nannoptera* increase their copulation latency after exposure to a rival, as also shown in the previous study on *D*. *pseudoobscura* [21], and exposure to a rival dramatically increased copulation latency in the related species *D*. *subobscura* [34,36]. One tentative explanation of this counterintuitive result is that the increase in latency might be due to increased “fatigue” of males caused by male-male interactions resulting in diminishing reproductive capacities of males. However, this remains speculative for *D*. *pseudoobscura*.

If the auditory cue removal trials are ignored, and the non-significant differences are accepted as probably representing a lack of detection of rival males, then the remaining trials fit a consistent pattern similar to that observed by Bretman et al. [13] (see Table 2). In *D*. *pseudoobscura* as in *D*. *melanogaster*, visual cues were less important than olfactory or tactile cues for allowing a male to alter copulation duration and latency after exposure to a rival male. However, in *D*. *pseudoobscura* the removal of olfactory or tactile cues alone appeared to be enough to prevent a male responding to a rival, whereas in *D*. *melanogaster* males only failed to respond to rivals when any two cues of tactile, olfactory, or auditory were removed. As the two members of the genus *Drosophila* in which cue use has been examined show different combinations of cues for detecting rivals, cue use must be evolutionarily labile. At present, we do not know how rapidly cue use can evolve. It would be interesting to examine the cues used in more closely related species, such as *D*. *pseudoobscura*’s sibling species *D*. *persimilis*, or close relatives of *D*. *melanogaster*. This evolutionary lability suggests that the use of cues in different species may have evolved in response to each species’ mating system and environment [1]. For example, *D*. *bifasciata* shows no increase in copulation duration after exposure to rivals [36], probably because this species lives at extremely high local densities, and males are unlikely to encounter a situation where rivals are not present. Unfortunately the mating ecology of wild *D*. *pseudoobscura* and *D*. *melanogaster* are not well understood, making it hard to determine exactly why *D*. *pseudoobscura* has evolved the more restrictive conditions for response to rivals. One possibility is that the costs of responding inappropriately to rivals are higher in *D*. *pseudoobscura*. Recent research has found that the benefits to male *D*. *melanogaster* of increasing copulation duration after exposure to a rival is relatively short lived, and that if a rival exposed male lives a long time the change in behaviour will actually reduce his fitness [25]. *D*. *pseudoobscura* may typically have a longer lifespan in nature than *D*. *melanogaster*, amplifying the costs of responding inappropriately. Evidence from within species suggests that higher latitude species and populations may typically have higher longevities, supporting this possibility [37]. Alternatively, rates of polyandry might be lower in *D*. *pseudoobscura*, making response to rivals less important than for *D*. *melanogaster*, or *D*. *pseudoobscura* males may often encounter heterospecific males making it important to avoid inappropriate responses.

**Table 2:** Comparison of male responses after treatment manipulations between *D*. *pseudoobscura* and *D*. *melanogaster*.

Treatment (cues removed)	*D*. *pseudoobscura*	*D*. *melanogaster*	Consensus in male response observed in both species
A	S	S	Yes
O	NS	S	**No**
T	NS	S	**No**
V	S	S	Yes
AO	S	NS	**No**
AT	S	NS	**No**
AV	S	S	Yes
OT	NS	NS	Yes
OV	NS	NS	Yes
TV	NS	S	**No**
AOT	S	NS	**No**
AOV	S	NS	**No**
ATV	NS	NS	Yes
OTV	NS	NS	Yes
AOTV	S	NS	**No**

Cues removed are Olfactory (O), Tactile (T), Visual (V) and Auditory (A). *D*. *melanogaster* data is taken from Bretman *et al*., [13]. Results are from comparisons of manipulated treatments to their corresponding internal controls, except in *D*. *pseudoobscura* Auditory treatments. In both cases, a significant result indicates the ability of rival perception. S and NS indicate significant and non-significant results respectively. See Table 1 for details of treatments.

Rival recognition often involves multiple cues in the same, or different, sensory modalities [35]. The use of several cues may be a way for animals to avoid recognition errors [12]. Detecting rival males and responding appropriately is likely to be important for males of a wide range of species, as competition over access to females is often a key aspect of male success [1, 38]. However, if responding to rivals is costly, then males should be selected to only respond when facing true rivals. Hence males might be expected to distinguish between effective and ineffective competitors. For example, it might be key to distinguish not only between males and females, but also conspecific males from heterospecifics, and immature, injured or subordinate males from mature rivals. This may be further complicated as rival males may often have conflicting interests in cue detection- avoiding detection by rivals may allow a male increased success if other males fail to respond to him. In many species, there are cryptic males that mimic females, and thereby avoid responses by rival males. The use of multiple cues might make it more difficult for rivals to remain cryptic. Studying cue use by males in classic “cryptic male” species, such as side blotched lizard (*Uta stansburiana*) [39] or ruffs (*Philomachus pugnax*) [40] might be particularly interesting as a result. Alternatively, a strong signal of being an effective rival might be important in gaining dominance over rivals. In species where males have clear signals they use in dominance interactions, such as the extremely long eye stalks of stalk-eyed flies [41], cues used to detect male rivals may be selected for particularly high detectability.

Cues are often highly evolutionarily labile (e.g. *Drosophila* courtship song components [42]). This lability, combined with potential selection for cues to evolve in divergent directions (e.g. stronger cues for dominance, female attraction, weaker cues to avoid conflict costs), and selection on all males to effectively detect rivals, has the potential to result in arms-races in cue presentation, detection and response between conflicting individuals. Similar arms races are already well studied in conflicts between males and females, predators and prey (e.g.[43]), and hosts and parasites (e.g.[44]). The ability to perceive and use several cues by males in *Drosophila* may be a way to maintain a low error rate in the face of continuous adaptation by rival males. In *D*. *melanogaster* and *D*. *pseudoobscura*, rival males may gain benefits from not being detected by other males through avoiding aggression, increasing female access and diminishing sperm competition for example. Tentative support for this rapid evolution of cue use comes from the result of Kim et al. [19] that male *D*. *melanogaster* only responded to rival exposure if they were able to see them, and that the response was due to males tracking the red eyes of rivals. This directly contradicts the results of Bretman et al. [13] who found that visual cues were irrelevant for male *D*. *melanogaster* response to rivals. Our results here also suggest that visual cues are unimportant for responding to rivals, albeit in *D*. *pseudoobscura*. The difference between the results of Bretman et al. [13] and Kim et al. [19] may be due to the use of different strains of *D*. *melanogaster*, with the teams using the Dahomey and Canton S strains respectively. If these results are to be trusted, this suggests that the cue use within *D*. *melanogaster* has evolved rapidly enough for two strains of the same species to use very different sets of cues. Further studies on additional strains, or on additional species would be useful in resolving this. In addition, recent work has questioned whether an over-reliance on easy to work with model species, such as *D*. *melanogaster* and *D*. *pseudoobscura*, might create a bias our understanding of biology [45]. As conflict between males over access to females is important for so many species, expanding studies of how cues are used to detect and respond to rivals should be a key goal of future research.

In summary, it has be shown that *D*. *pseudoobscura* use a combination of sensory cues to detect rival males and respond by adjusting their copulation duration. These results broadly follow previous work in *D*. *melanogaster* ([13]; see Table 2), but differ in the details, suggesting that the suite of cues used to detect rivals is evolutionarily labile, and likely to have evolved in response to the mating system and reproductive ecology of each species.
